# What Is a Disease?

**Published:** 1886-07

**Authors:** 

**Affiliations:** Editor and Publisher of “Problems of Nature”


### WHAT IS A DISEASE?

BY H B. PHILBROOK.
Editor and Publisher of "Problems of Nature."

Every disorder of the body that is not a wound or caused by a substance that poisons is called a disease, and a clear description of what actually constitutes a disease is not found in the works upon medicine. A disease is only a commencement of a process of destruction of the body by what is called decomposition. It is the commencement of the death of the body. A destruction of the cells and tissues of the body by a force that is in the organization is the actual character of a disease. The other complaints of the body are either a mere weakness or an injury by a substance not connected with the body.

A power that constructs and operates the organization is capable of giving death a defiance, and preventing any possession of the system by a disease of any character, if a punishment or a death of the person is not wanted by a controller of a universe of affairs. Every actual disease is either a punishment for a bad practice or habit, or it is given for the death of the person. Only a question of the use a person can afford a world of mortals is considered in the bestowal of a disease to one, and it is for a punishment or death according to the use a person can give a community. It is always for a placing of the person in a position to be of use when the disease is adopted by a power of all creation. If the person is absolutely useless, he or she is given a call for a sphere where a use can be obtained of the soul of the person. Only a few of such worthless persons are permitted to stalk about a city or town, and their use is to afford a whole community examples of worthlessness and degradation. All the balance of the class are passed to a graveyard in a proper day of their worthlessness.

If the person is capable of being made useful by a destruction of a bad practice or habit, a disease is but a temporary affair, and all the physicians are given credit for a cure of such cases.

When a cure is not effected, a doctor is scolded for the death of the person. A physician is as worthless to cure a disease that is given for the death of a person as he is to give a halt to a flow of a river, and it is the case with every physician that every kind of disease is incurable in some instances. A discovery of the cause of this is what the medical profession wants.

A cause can be found on a searching of the instances of the casting out of devils by a Saviour in the days of his advent, and in the obsession of persons noticed by all people in all the days of the past, and by the spiritualist in every case of the control of a person by a spirit of a person who was not long dead.

Every disease is a copy of one a person of the family or a person interested in the family died of before the patient was attacked. A giving of a disease by a soul of a departed mortal, is a plan of a Creator for chastising a person of bad habits, as giving death to one who is wholly useless.

This astonishing fact is going to be observed by all people in a short time. A drug is given dismissal when a cause and cure of disease is known.

